# Measurement and implications of the distance between the sphenopalatine ganglion and nasal mucosa: a neuroimaging study

**DOI:** 10.1186/s10194-018-0843-5

**Published:** 2018-02-13

**Authors:** Joan Crespi, Daniel Bratbak, David Dodick, Manjit Matharu, Kent Are Jamtøy, Irina Aschehoug, Erling Tronvik

**Affiliations:** 1 Department of Neurology, St Olav’s University Hospital, Edvards Grieg’s gate 8, 7030 Trondheim, Norway; 2 Department of Neuromedicine and Movement Science, NTNU (University of Science and Technology), Trondheim, Norway; 3 Norwegian Advisory Unit on Headaches, Trondheim, Norway; 4 Department of Neurosurgery, St Olav’s University Hospital, Trondheim, Norway; 5 Department of Neurology, Mayo Clinic, Phoenix, AZ USA; 6 National Hospital of Neurology and Neurosurgery, London, UK; 7 Department of maxillofacial surgery, St Olav’s University Hospital, Trondheim, Norway

**Keywords:** Sphenopalatine ganglion, Pterygopalatine ganglion, Local anaesthetics, Intranasal, Block

## Abstract

**Background:**

Historical reports describe the sphenopalatine ganglion (SPG) as positioned directly under the nasal mucosa. This is the basis for the topical intranasal administration of local anaesthetic (LA) towards the sphenopalatine foramen (SPF) which is hypothesized to diffuse a distance as short as 1 mm. Nonetheless, the SPG is located in the sphenopalatine fossa, encapsulated in connective tissue, surrounded by fat tissue and separated from the nasal cavity by a bony wall. The sphenopalatine fossa communicates with the nasal cavity through the SPF, which contains neurovascular structures packed with connective tissue and is covered by mucosa in the nasal cavity. Endoscopically the SPF does not appear open. It has hitherto not been demonstrated that LA reaches the SPG using this approach.

**Methods:**

Our group has previously identified the SPG on 3 T–MRI images merged with CT. This enabled us to measure the distance from the SPG to the nasal mucosa covering the SPF in 20 Caucasian subjects on both sides (*n* = 40 ganglia). This distance was measured by two physicians. Interobserver variability was evaluated using the intraclass correlation coefficient (ICC).

**Results:**

The mean distance from the SPG to the closest point of the nasal cavity directly over the mucosa covering the SPF was 6.77 mm (SD 1.75; range, 4.00–11.60). The interobserver variability was excellent (ICC 0.978; 95% CI: 0.939–0.990, *p* < 0.001).

**Conclusions:**

The distance between the SPG and nasal mucosa over the SPF is longer than previously assumed. These results challenge the assumption that the intranasal topical application of LA close to the SPF can passively diffuse to the SPG.

## Background

The sphenopalatine ganglion (SPG) has been a target for treatment of headache disorders for more than a century [1]. Different approaches, including the direct application of pharmacological substances or neurolysis, have been used in an attempt to block the sphenopalatine ganglion to treat a broad range of headache and facial pain disorders such as cluster headache, migraine, trigeminal neuralgia, postherpetic trigeminal neuralgia, post-traumatic headache, post-dural puncture headache, and hemicrania continua [2, 3]. Attempts at pharmacological blockade include direct percutaneous injections towards the SPG or topical intranasal administration. While it appears reasonable to posit that a substance will reach the SPG with an image-guided injection, the ability of a substance to passively diffuse and reach the SPG after intranasal application is uncertain. This is especially true since there is not readily available clinical biomarker to verify that the target (SPG) has been engaged and blocked.

The first RCT evaluating the intranasal administration of local anaesthetics (INALA) was published in 1996 demonstrated a significant acute treatment effect in patients with migraine [4]. In 1999 these results were confirmed in a second RCT by the same group [5], resulting in a Level C recommendation for INALA for acute migraine treatment [6]. Both studies hypothesized that the mechanism of action for INALA is neural blockade of the SPG. In order to reach the SPG, the local anaesthetics (LA) must diffuse from the intranasal cavity. The authors argue that this is reasonable since the SPG is ≤ 1 mm below the nasal mucosa in the area of the sphenopalatine foramen (SPF), citing the work of Sluder from 1909 [7]. In line with this hypothesis and evidence base, the application of intranasal LA as close as possible to the SPF became a widely adopted procedure in clinical practice and drove the commercial development, marketing, and availability of intransal catheter devices designed to provide application of LA near the SPF.

Advanced imaging techniques allows the opportunity to determine the actual distance from the SPF to the SPG in living subjects. The aim of this study is to measure the distances between the nasal mucosa over the SPF and the SPG in 20 (40 sides) patients on fused MRI/CT images. We also review the literature on the efficacy of INALA for the treatment of headache and discuss the evidence that a drug applied intranasally over the SPF will freely diffuse to and engage the SPG.

## Methods

Our investigation has formerly identified the SPG on MRI in living humans [8]. In this study, the relative location of the SPG to bony landmarks in radiological images (fusioned CT and MRI images) was compared and found to be equivalent to the distances obtained in an anatomical cadaveric study by Keller [9]. By using the same image sets we were able to measure the distance from the nasal mucosa covering the SPF to the SPG on 20 living humans (*n* = 40 ganglia). The distance was measured by two physicians (JC, DFB). The 20 patients included in this study had been formerly included in two other trials where they underwent a block of the SPG using a new neuronavigation technique at St. Olavs Hospital, Trondheim, Norway, between October 2013 and February 2016. Ten patients had intractable chronic cluster headache [10] and ten patients had intractable chronic migraine [11]. All patients were examined with CT and MRI scans covering the region of the sphenopalatine fossa and neighboring regions. None of the patients eligible for inclusion were excluded. None of the patients had received previous injections towards the SPG, which might have altered the anatomy of the sphenopalatine fossa.

MR scans were performed on a 3 T scanner (Magnetom Skyra, Siemens, Germany). Technical parameters were as follows: Sagittal T2 weighted: Repetition time (TR) range 3780, echo time (TE) 111, slice thickness 2 mm, matrix 0.4 × 0.4 × 2.0 mm, field of view (FOV) 210, number of acquisitions 3; sagittal T1 weighted: TR range 710, TE 10, slice thickness 2 mm, matrix 0.4 × 0.4 × 2.0 mm, FOV 210, number of acquisitions 2; axial T2 weighted: TR range 4160, TE 110, slice thickness 2 mm, matrix 0.4 × 0.4 × 2.0 mm, FOV 220, number of acquisitions 2; and axial T1 weighted: TR range 710, TE 7.9, slice thickness 2 mm, matrix 0.4 × 0.4 × 2.0 mm, FOV 210, number of acquisitions 2. All CT scans were performed using a helical CT scanner (Somatom sensation 64, Siemens, Germany) set at effective mAs 63, 120 kV, slice thickness1 mm, reconstruction increment 0.7 mm, collimation 12 × 0.6 mm, Kernel U 70, window width 450 HU and window centre 50 HU. Fusion of MR and CT images was performed using Brainlab iPlan 3.0 (Brainlab AG, Feldkirchen, Germany).

Both studies were approved by the regional ethics committee (ref. 2012/164 and 2014/962), the Norwegian Medicines Agency (EUDRACT nr: 2012–000248-91 and 2014–001852-43) and registered at ClinicalTrial.gov (NCT02019017 and NCT02259075). Written informed consent was obtained from all patients.

The SPG was localized in T2 weighted images. The closest point of the nasal mucosa covering the SPF was localized on CT-scan images and not in MRI in order to reduce the partial volume effect.

### Statistical analysis

SPSS version 24.0 (SPSS Inc., Chicago, Illinois, USA) was used in the data analyses.

Data distributions were expressed as means and standard deviations (SD), results are given as mean ± standard deviation if not otherwise stated. Interobserver variability was evaluated using the intraclass correlation coefficient (ICC). A post hoc analysis for intra-individual variability was assessed using an independent samples t-test.

## Results

Table 1 illustrates the demographic characteristics of the 20 patients examined in this study.

**Table 1:** Demographics of the sample

	All patients (*n* = 20)
Number of females/males	15/5
Mean age, years ± SD (range)	44.8 ± 13.0 (24–68)
Number of Caucasians	20/20
Primary headache	20/20
• Chronic cluster headache	10/20
• Chronic migraine	10/20

The mean distance from the SPG to the closest point of the nasal cavity directly over the mucosa covering the SPF was 6.77 mm (SD 1.75; range, 4.00–11.60). The interobserver variability was excellent (ICC 0.978; 95% CI: 0.939–0.990, *p* < 0.001). There was no significant difference between the average distances in the right and left sides, with a mean difference right-left of − 0.58 mm (95% CI: -1.76-0.60, *p* = 0.327).

**Fig. 1:**
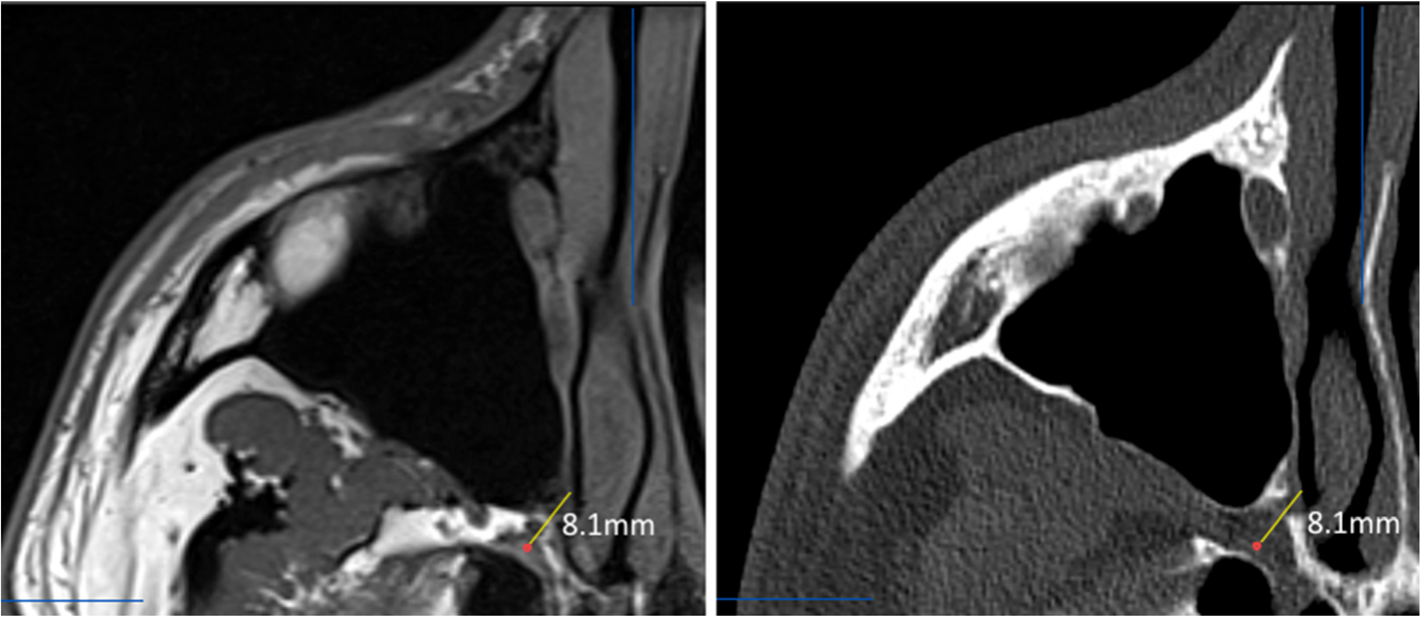
Axial images through the SPG in one of the patients. Left: T1 weighted MRI. Right: CT scan. Both images show the same anatomical plane. The SPG (red dot) is first localized in the MRI scan and the closest point of the nasal mucosa through the SPF is localized in fusioned CT images. In this example, the distance was 8.1 mm (yellow line). Notice the typical crescent form of the SPG anterior to the opening of the Vidian canal

The SPG was localized in MRI scans in all patients. Fig. 1 shows axial images (T1 weighted MRI and CT) through the SPG in one of the patients of the study.

## Discussion

This is the first study to measure the distance between the mucosa overlying the SPF and the SPG in living humans. The mean distance of 6.77 mm is higher than the distance described in cadaveric studies, possibly as a result of dessication of post-mortem tissue.

It has been assumed that a LA applied intranasally in the proximity of the SPF can reach the SPG [1, 4, 5, 12, 13]. An important prerequisite for such a hypothesis is that the distance between the surface of the nasal mucosa and the SPG is sufficiently short. Sluder estimated the distance to be as little as 1 mm and this has been cited among many advocating for the therapeutic effect of INALA [7]. However, Sluder also acknowledged that the SPG may rest up to 9 mm from the SPF and that there is considerable variability between individuals [7]. Unfortunately, the methodology used to assess the localization of the SPG was not described nor was the size or demographics of the sample defined. Penteshina analysed 70 SPG and found significant individual differences in the structure and topography of the SPG [14]. SPG’s size was stable (3 to 5 mm) but its position in relation to the anterior foramen of the Vidian canal, SPF, palatine bone and maxillary nerve were variable. In this study, the SPG was located 3–4 mm from the nasal mucosa membrane in 35 cadavers, but in 20 cases, it was at a depth of 10 mm and surrounded by fatty tissue. In some cases, the SPG was located in the Vidian canal making the SPG inaccessible to INALA [14]. Only in 15 out of 70 ganglia was the SPG closely adjacent to the nasal mucosa membrane [14].

**Fig. 2:**
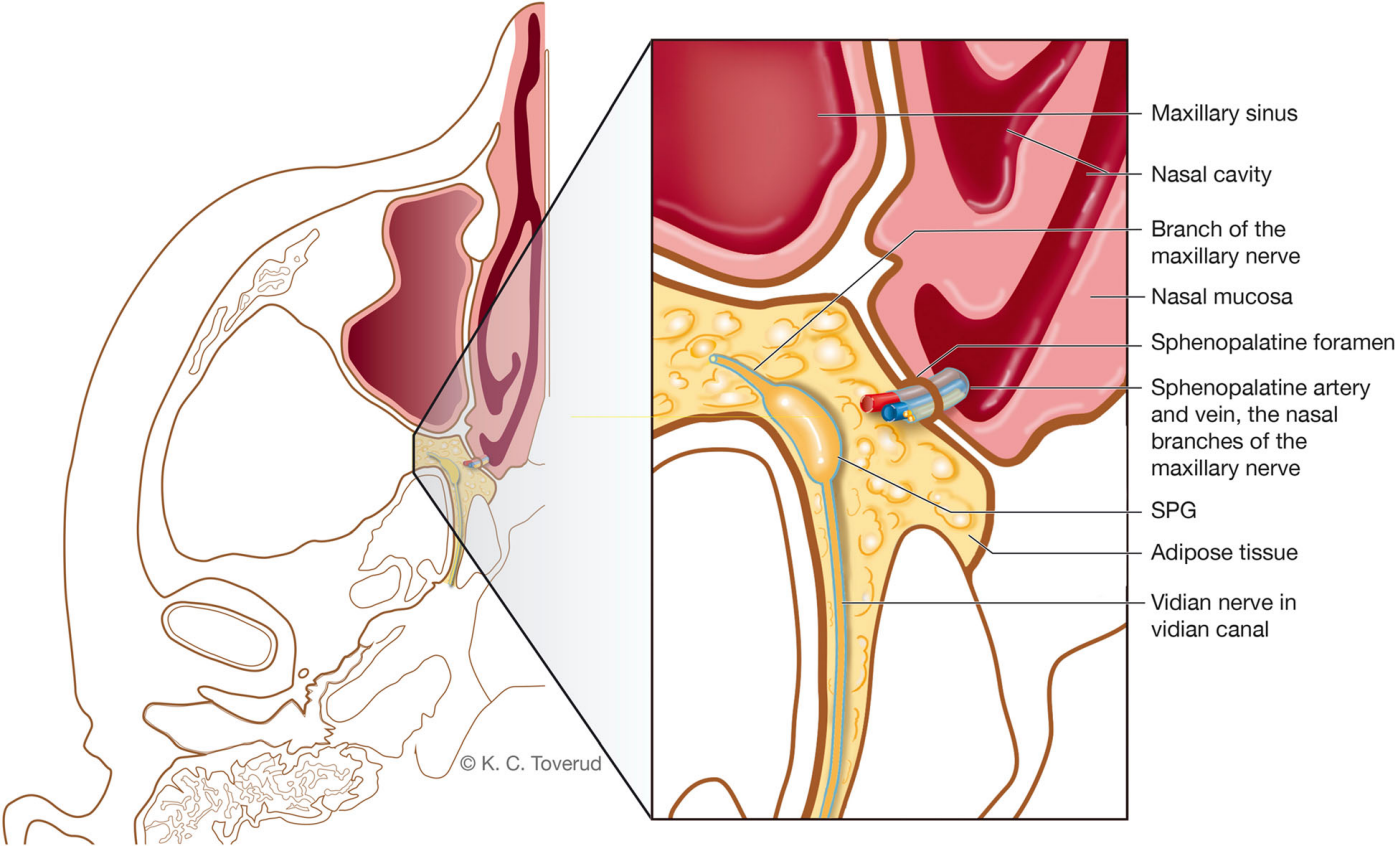
Illustration of the relation between the nasal cavity and the sphenopalatine fossa (axial plane). In order to reach the SPG, a drug applied intranasally over the sphenopalatine foramen will have to diffuse through mucosa, the sphenopalatine foramen, which is packed with neuro-vascular structures and connective tissue, and the fat tissue filling the sphenopalatine fossa. SPF: sphenopalatine foramen; SPG: sphenopalatine ganglion

In addition to the distance between the nasal mucosa and the SPG, there are several barriers through which LA must diffuse through to reach the SPG, including nasal mucosa; neurovascular structures connective tissue filling the SPF and adipose tissue in the sphenopalatine fossa between the SPF and the SPG (Fig. 2).

**Fig. 3:**
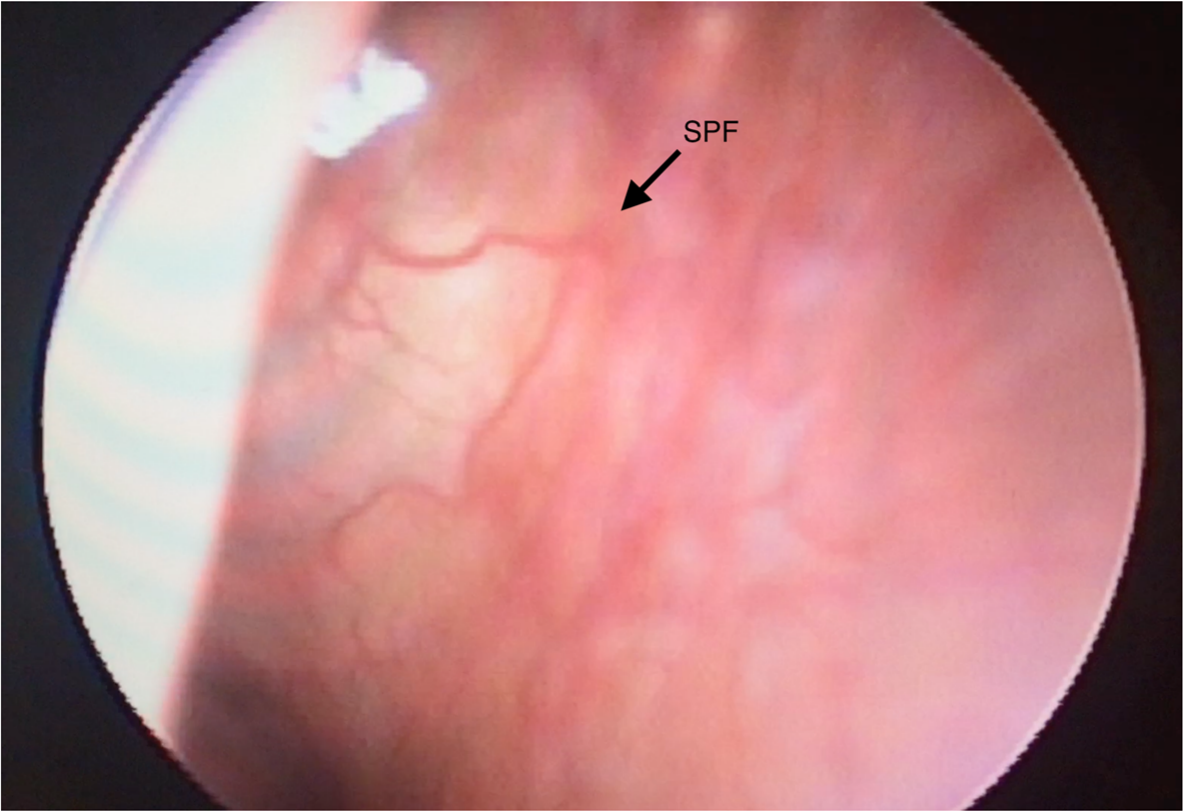
Rhinoscopy showing the mucosa over the sphenopalatine foramen (SPF) and the sphenopalatine artery (arrow). The SPF does not appear as an open foramen communicating directly with the sphenopalatine fossa. The SPF is covered by mucosa and packed with neurovascular structures and connective tissue

The nasal cavity and the sphenopalatine fossa are divided by the vertical wing of the palatine bone with neurovascular structures entering and exiting the nasal cavity through the SPF. The SPF is covered by mucosa and it does not present as an open foramen that communicates with the sphenopalatine fossa (Fig. 3). Since LA cannot transverse through bone, it has to pass through the foramen alongside the vascular structures. LA entering the vascular structures may enter the systemic circulation and be transported away from the SPG. Some studies raise the question whether the observed effect of INALA might be due to systemic absorption of the anesthetic rather than a block of the SPG [15–17]. After passing through the SPF, LA would enter the sphenopalatine fossa, which is filled with adipose tissue requiring diffusion of the LA through adipose and connective tissue in order to reach the SPG.

Rusu et al. performed dissections of the sphenopalatine fossa in 20 human cadavers and observed that 30% of the SPGs did not appear as single macroscopic structures, but had two distinctive partitions (one superior and one inferior) [18]. In addition, all patients had neuronal clusters and neuronal cords within the proximity of the SPG (intrinsic intraneural dispersed sphenopalatine microganglia), which were not apparent macroscopically. These factors may account for the therapeutic failure observed in some patients undergoing procedures targeting the SPG [18, 19].

The SPF lies lateral in the wall of the nasopharynx, which constitutes an anatomical challenge when trying to gain access to it. Some authors have emphasized the importance of a proper technique to achieve a transnasal block of the SPG [5, 20], particularly that the patient’s head is properly extended and rotated 30 degrees towards the desired side.

Most commercially available catheters do not visually localize the SPG, either through endoscopy or fluoroscopy. The blind application of LA may therefore not approximate the SPF. Alherabi et al. dissected 16 lateral nasal walls and documented that the distance from the nasal sill to the SPF varies widely from 55 to 76 mm and the range of the angle of elevation formed between the SPF to the nasal sill is 11–12 degrees [21]. The authors describe that the standard reference points to localize the SPF are widely different and of little practical help. Other groups have also described the anatomical variation of the SPF [22]. The size of the foramen is also variable. Prades et al. measured the SPF in 12 skulls and reported a mean height of 6.1 mm (5.2–6.8 mm) and a mean width of 2.5 mm (2.4–2.5 mm) [23]. In most approaches, LA are applied to a larger area within the nasal cavity and therefore the concentration would have to be high to allow enough substance to diffuse close to the SPG.

### Rationale for LA block of the SPG

**Fig. 4:**
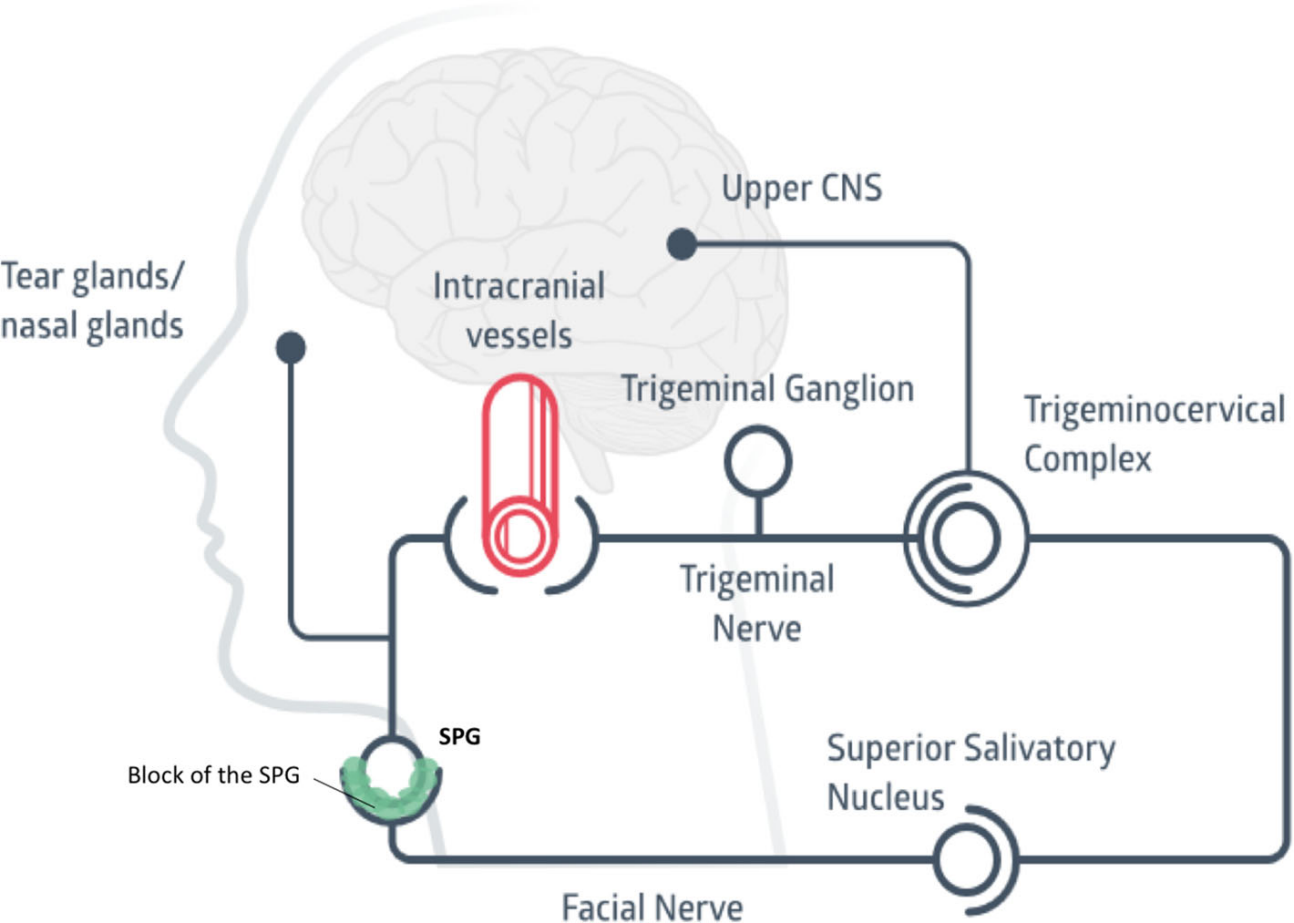
Diagram showing the involvement of the sphenopalatine ganglion (SPG) in the physiopathology of trigeminoautonomic headaches. The afferent part of this loop is mediated by the trigeminal nerve, which sends nociceptive signals from the dural blood vessels to the trigeminocervical complex. This information projects to higher brain structures, resulting in cephalic pain. The efferent part of this loop conveys mostly through the superior salivatory nucleus, exiting the brain stem via the facial nerve and reaching the sphenopalatine ganglion through the greater petrosal nerve. Postganglionic fibres exit the sphenopalatine nerve towards the dural vessels, closing the loop. Blocking the SPG might reduce the afferent input of signals towards the trigeminal system and reduce the activation of the trigeminocervical complex. CNS: central nervous system

The trigemino-autonomic reflex, where parasympathetic efferents with synapses in the SPG activate meningeal trigeminal nociceptors, is thought to be important in several headache conditions [24]. A postulated mechanism to understand why blocking the SPG may be effective is by reducing the efferent release of neuropeptides on dural nociceptors and thereby reducing afferent trigeminovascular activity (Fig. 4).

### Alternative mechanisms of action

The positive effect of the transnasal topical block in headache shown in several studies could be due to a trigeminal block rather than a block of the SPG. This possible mechanism has already been suggested in cluster headache by Barre [20], Raskin [25] and Robbins [26]. Barre proposed several possible mechanisms of action of cocaine in cluster headache: local blockade of neural transmission of nerves in the vicinity of the Vidian Nerve, SPG or maxillary division of the trigeminal nerve; via its sympathomimetic effects in inducing local or regional vasoconstriction secondary to the development of sensitization to catecholamines; or a combination of both of the above [20]. Later observations that cocaine and lidocaine appear to be similarly effective in cluster headache led to some authors favoring the anesthetic effect over the vasoconstrictor hypothesis [27].

It is known that applying a local anesthetic intranasally blocks first and second trigeminal nerve endings in the nasal mucosa, which is a standard technique used in transnasal surgery. Hardebo et al. described in a series of 24 patients that when intranasal lidocaine was effective, pain was usually reduced in the orbital and nasal region [27]. Schueler et al. described that the maxillary and mandibular branches of the trigeminal nerve in rats have both intra and extracranial receptive fields and that its stimulation could release CGRP [28]. Thus, one cannot exclude a trigeminal block or systemic absorption as a possible explanation for the observed effect.

### Review of evidence on intranasal administration of LA in headache

Most studies evaluating the administration of topical intranasal LA are of poor quality. A total of 9 RCTs have been conducted using INALAs (Table 2). Three were negative for the primary endpoint [29–31]. While these studies have claimed that the SPG was the target, it is unclear whether the study drug reached the SPG. Even though most of the procedures administrating intranasal LA are well tolerated and considered to be safe, adverse events have been reported [2]. The most frequently reported serious adverse event is epistaxis. Most of the adverse events are transient and include bitter taste, oropharyngeal numbness, ipsilateral nostril and eye burning sensation, nasal discomfort, diplopia and reduced buccal opening. Cady et al. using the Tx360® catheter, found that the most common side effects were lacrimation, unpleasant taste and mouth numbness [13, 32].

**Table 2:** Summary of studies evaluating topical intranasal administration of LA in pain disorders of the head and face

Author	Drug	Concen-tration	Volume	Condition	RCT	Effect	Nr of patients	Technique	Ref.
Sluder	Cocaine	4–70%	A drop	Meckel’s ganglion neuralgia	No	Positive effect in a series of patients	5	Applicator, surgery	[7]
	Alcohol	–	A drop						
	formaldehyde	0,4%	N/S						
	Silver nitrate	2%	N/S						
Barre	Cocaine	5–10%	N/S	CH	No	Positive effect in a series of patients	11	Barre’s technique^a^	[20]
Kittrelle	Cocaine	5%	–	CH (NTG-induced)	No	> 75% reduction in headache intensity within 3 min, in 4 of 5 patients with lidocaine	5	Barre’s technique^a^	[12]
	lidocaine	4%	1 ml						
Hardebo	Cocaine	10%	0,3 ml	CH	No	Lidocaine and cocaine equally effective	24	Nasal droper	[27]
	Lidocaine	4%	0,5–0,8 ml						
Robbins	Lidocaine	4%	4–6 sprays	CH	No	54% mild to moderate relief after treatment	30	Spray	[26]
Kudrow	Lidocaine	4%	0,4 ml	Migraine	No	Migraine attacks aborted in 12 of 23 patients	23	Barre’s technique^a^	[38]
Maizels	Lidocaine	4%	0,5 ml	Migraine	Yes	55% of patients that received lidocaine had at least 50% reduction of headache within 15 min (primary endpoint)	81	Barre’s technique^a^	[4]
Maizels	Lidocaine	4%	0,5 ml	Migraine	Yes	Randomized trial with open-label follow-up. Controlled trial: 35.8% of patients had headache relieved to mild or none 15 min. After treatment.	131	Barre’s technique^a^	[5]
Maizels	Lidocaine	4%	0,5 ml	Migraine	No	Prevention of the development of headache following aura.	1	Barre’s technique^a^	[39]
Saberski	Lidocaine	20%	N/S	Postherpetic neuralgia	No	Decrease of the pain (therapy repeated 11 times)	1	Applicator dipped in anaesthetic	[40]
Costa	Lidocaine	10%	1 ml	CH (NTG-induced)	Yes	All patients responded to both anaesthetics with complete cessation of induced pain (31.3 ± 13.1 min for cocaine and 37.0 ± 7.8 for lidocaine. For saline, pain severity increased initially and resolved with a latency of 59.3 ± 12.3 min.	15	Cotton swab under rhinoscopy	[34]
	Cocaine	10%	1 ml						
Blanda	Lidocaine	4%	1 ml	Migraine	Yes	The study was negative for the main outcome measure (decrease of ≥50% of initial pain score or an absolute pain score ≤ 2.5 cm at 5 min.	49	Barre	[29]
Windsor	Lidocaine	2%	1–2 ml	Herpes keratitis	No	Relief of pain in one case report	1	Applicator developed by the authors	[15]
Chae	Lidocaine	2%	N/S	Post-traumatic headache	No	Reduction of VAS scale within 15 min in both patients (from 8/10 to 0/10 in the first and from 10/10 to 2/10 in the second)	2	N/S	[41]
Cohen	Lidocaine	4%	N/S	Postdural puncture headache	No	Relief of pain in a series of patients	13	Applicator saturated	[42]
Bakbak	Lidocaine	10%	2 ml	CH	No	Relieve of pain and autonomic symptoms	1	Cotton-tipped applicator	[43]
Pfaffenrath	Lidocaine	6%	0,1 ml	Migraine	Yes	Primary endpoint not met: proportion of pain-free patients two hours after treatment. Improvement of several secondary endpoints.	140	Spray	[30]
	Ketorolac		0,1 ml						
Candido	Ropivacaine	0,5%	0,5 ml	1 TN, 1 CM, 1 post-herpetic neuralgia	No	All 3 patients reported pain relief within the first 15 min. Post-treatment.	3	Tx360®	[44]
	Dexamethasone	–	–						
Cady	Bupivacaine	0,5%	0,3 ml	Chronic migraine	Yes	Reduction of pain compared to placebo at 15 min, 30 min and 24 h compared to placebo (primary endpoint). Decreased HIT-6 score compared to placebo at 1 and 6 months.	38	Tx360®	[30]
Mohammadkarimi	Lidocaine	10%	1 puff	Acute headache	Yes	Significant reduction of mean pain scores at 1 min (primary endpoint). The effect was sustained at 30 min	90	Spray	[37]
Cohen	Lidocaine	5%	N/S	Postdural puncture headache	No	Relief of pain in a series of patients	32	Applicator saturated	[45]
Cady	Bupivacaine	0,5%	0,3 ml	Chronic migraine	Yes	Primary endpoint: statistically significant reduction of NRS scores. A comparison of the number of headache days during the baseline period and 1 month post-treatment was not significant.	38	Tx360®	[32]
Schaffer	Bupivacaine	0,5%	0,3 ml	Acute headache	Yes	Primary endpoint (50% reduction in pain at 15 min) negative.	93	Tx360®	[31]
Androlaukis	Bupivacaine	0,5%	0,6 ml	Hemicrania continua	No	Reduction in average intensity and frequency of headaches and autonomic symptoms.	1	Tx360®	[3]
Dance	Lidocaine	4%	N/S	Migraine (pediatric patients age 7–18)	No	Reduction of pain scores (only abstract available).	85	Allevio®	[46]

*LA* local anesthetic, *SPF* sphenopalatine foramen, *RCT* randomized clinical trial, *CH* cluster headache, *N/S* not specified, *TN* trigeminal neuralgia, *CM* chronic migraine, *NTG* nitroglycerine

^a^Barre’s technique: the patient lies supine with extended neck (45 degrees) and head rotated 30 degrees ipsilateral to the pain. After the desired volume of anesthetic is applied, the patient should stay in the described position for 30 s. [3–5, 7, 12, 13, 15, 20, 26, 27, 29–32, 34, 37–46]

When one applies LA intranasally, most of the volume will descend to the pharynx and the patient will often swallow the fluid, commonly complaining of a bitter taste after the procedure [15]. As a consequence, the final volume of LA that will remain on the surface of the SPF to passively diffuse to the SPG is likely to be small. The bitter taste of most LA constitutes a problem for blinding. This issue has not been properly assessed and might constitute an important bias in several studies.

### Different techniques for intranasal administration of LA

The technique which has been most commonly used is the one described by Barre [20] (Table 2). New intranasal catheters that claim to offer higher tolerability have been developed [2]. Other indirect and blind techniques have been described for the use of INALA [15, 26, 33–35, 30, 36, 37] but the same limitations and anatomical restrictions discussed above still apply.

### Limitations

Some limitations of the study are its relatively small sample (20 subjects) and that the gender ratio is skewed (m:f 1:3). All examined patients are Caucasians. The method used in this article to identify the SPG on MRI images [8] is not an established method. The presence of autonomic symptoms pre- and posttreatment was not recorded in these group of patients.

### Future perspectives

Different studies have used different LA, or combinations of them. The concentration of the LA also varies across the studies (Table 2). Some have mixed LA with corticosteroids or with other analgesics. Different LA have different pharmacological properties that might influence their ability to reach the SPG by free diffusion. The volumes of local anesthetic have varied between 0.3 and 2 ml. Such important aspects as which drug (or combination), which concentration and volume would be most suitable, have not been properly assessed in the literature. The technique used to apply the LA in the proximity of the SPF varies in the different studies. Further studies that assess the pharmacological and anatomical basis to support that a drug applied over or in the proximity of the SPF, will actually reach the SPG by free diffusion, are warranted.

## Conclusion

SPG blockade through the intranasal injection of LA has been employed widely as an acute and preventive treatment for a variety of primary and secondary headache disorders. However, the evidence is mixed and inconclusive. The rationale to justify this approach has been the assumption that the SPG lies directly under the nasal mucosa. In this study on living humans, we show that the distance between the SPG and the nasal mucosa over the SPF is significantly longer than previously assumed. Moreover, the bony and mucosal anatomy, combined with the connective and adipose tissue that fills the sphenopalatine fossa, challenge the assumption that intranasal topical application of LA may reach the SPG. Whether these anatomical considerations discussed above have clinical implications is not known. Further research using biomarker evidence to confirm whether the SPG has been blocked after the local intranasal application of LA, and high quality RCTs with adequate placebo to protect the blind, are necessary to assess the veracity and efficacy of this procedure.

### Clinical implications

- The distance from the nasal mucosa to the sphenopalatine ganglion (SPG) appears to be longer than previously assumed.
- 3 of 9 RCTs where local anaesthetics were applied intranasally have been negative. These studies have claimed that the SPG was the target but this has not been proved.
- Further studies that assess the pharmacological and anatomical basis to support that a drug applied in the proximity of the sphenopalatine foramen, will actually reach the SPG by free diffusion, are warranted.

## Abbreviations

CH: Cluster headache
CM: Chronic migraine
CT: Computerized tomography
INALA: Intranasal administration of local anaesthetics
LA: Local anesthetic
MRI: Magnetic resonance images
N/S: Not specified
NTG: Nitroglycerine
RCT: Randomized clinical trial
SPF: Sphenopalatine foramen
SPG: Sphenopalatine ganglion
TN: Trigeminal neuralgia

## References

[1] Sluder G (1908) The role of the sphenopalatine ganglion in nasal headaches. AR Elliott Publishing Company. N Y State J Med 27:8–13.

[2] Robbins MS, Robertson CE, Kaplan E, Ailani J, Charleston L, Kuruvilla D (2016). The sphenopalatine ganglion: anatomy, pathophysiology, and therapeutic targeting in headache. Headache.

[3] Androulakis XM, Krebs KA, Ashkenazi A (2016). Hemicrania continua may respond to repetitive sphenopalatine ganglion block: a case report. Headache.

[4] Maizels M, Scott B, Cohen W, Chen W (1996). Intranasal lidocaine for treatment of migraine: a randomized, double-blind, controlled trial. JAMA.

[5] Maizels M, Geiger AM (1999). Intranasal lidocaine for migraine: a randomized trial and open-label follow-up. Headache.

[6] Marmura MJ, Silberstein SD, Schwedt TJ (2015). The acute treatment of migraine in adults: the american headache society evidence assessment of migraine pharmacotherapies. Headache.

[7] Sluder G (1909). The anatomical and clinical relations of the sphenopalatine (Meckel’s) ganglion to the nose and its accessory sinuses. N Y Med J.

[8] Bratbak DF, Folvik M, Nordgard S, Stovner LJ, Dodick DW, Matharu M, Tronvik, E (2017) Depicting the pterygopalatine ganglion on 3 Tesla magnetic resonance images. Surg Radiol Anat. 10.1007/s00276-017-1960-6 10.1007/s00276-017-1960-6 29274037

[9] Keller H (1980) Über Die Hintere Pfortenregion Der Fossa Pterygopalatina Und Die Lage Des Ganglion Pterygopalatinum. Doctoral Dissertation, Julius-Maximilans-Universitäts Würburg. 689340

[10] Bratbak DF, Nordgard S, Stovner LJ, Linde M, Folvik M, Bugten V (2016). Pilot study of sphenopalatine injection of onabotulinumtoxinA for the treatment of intractable chronic cluster headache. Cephalalgia.

[11] Bratbak DF, Nordgard S, Stovner LJ, Linde M, Dodick DW, Aschehoug I, Tronvik, E (2017) Pilot study of sphenopalatine injection of onabotulinumtoxinA for the treatment of intractable chronic migraine. Cephalalgia, 37(4):356-64 10.1177/0333102416648328 PMC5394468 27154997

[12] Kittrelle JP, Grouse DS, Seybold ME (1985). Cluster headache. Local anesthetic abortive agents. Arch Neurol.

[13] Cady R, Saper J, Dexter K, Manley HR (2015). A double-blind, placebo-controlled study of repetitive transnasal sphenopalatine ganglion blockade with tx360((R)) as acute treatment for chronic migraine. Headache.

[14] Penteshina, NA (1965) Morphology of the Pterygopalatine Ganglion. Zh Nevropat Psikhiat 65(9):1325–30. 5871754

[15] Windsor RE, Jahnke S (2004). Sphenopalatine ganglion blockade: a review and proposed modification of the transnasal technique. Pain Physician.

[16] Ruskin AP (1979). Sphenopalatine (nasal) ganglion: remote effects including “psychosomatic” symptoms, rage reaction, pain, and spasm. Arch Phys Med Rehabil.

[17] Berger JJ, Pyles ST, Saga-Rumley SA (1986). Does topical anesthesia of the sphenopalatine ganglion with cocaine or lidocaine relieve low back pain?. Anesth Analg.

[18] Rusu MC, Pop F, Curca GC, Podoleanu L, Voinea LM (2009). The pterygopalatine ganglion in humans: a morphological study. Ann Anat.

[19] Gregoire A, Clair C, Delabrousse E, Aubry R, Boulahdour Z, Kastler B (2002). CT guided neurolysis of the sphenopalatine ganglion for management of refractory trigeminal neuralgia. J Radiol.

[20] Barre F (1982). Cocaine as an abortive agent in cluster headache. Headache.

[21] Alherabi A, Marglani O, Herzallah IR, Shaibah H, Alaidarous T, Alkaff H (2014). Endoscopic localization of the sphenopalatine foramen: do measurements matter?. Eur Arch Otorhinolaryngol.

[22] Scanavine AB, Navarro JA, Megale SR, Anselmo-Lima WT (2009). Anatomical study of the sphenopalatine foramen. Brazilian J Otorhinolaryngology.

[23] Prades JM, Asanau A, Timoshenko AP, Faye MB, Martin C (2008). Surgical anatomy of the sphenopalatine foramen and its arterial content. Surgical Radiologic Anatomy : SRA.

[24] Akerman S, Holland PR, Lasalandra MP, Goadsby PJ (2009). Oxygen inhibits neuronal activation in the trigeminocervical complex after stimulation of trigeminal autonomic reflex, but not during direct dural activation of trigeminal afferents. Headache.

[25] Raskin, NH (1988) The Hypnic Headache Syndrome. Headache: the journal of head and face pain 28(8):534–36. 10.1111/j.1526-4610.1988.hed2808534.x 3198388

[26] Robbins L (1995). Intranasal lidocaine for cluster headache. Headache.

[27] Hardebo JE, Elner A (1987). Nerves and vessels in the pterygopalatine fossa and symptoms of cluster headache. Headache.

[28] Schueler M, Messlinger K, Dux M, Neuhuber WL, De Col R (2013). Extracranial projections of meningeal afferents and their impact on meningeal nociception and headache. Pain.

[29] Blanda M, Rench T, Gerson LW, Weigand JV (2001). Intranasal lidocaine for the treatment of migraine headache: a randomized, controlled trial. Acad Emerg Med Off J Soc Acad Emerg Med.

[30] Pfaffenrath V, Fenzl E, Bregman D, Farkkila M (2012). Intranasal ketorolac tromethamine (SPRIX(R)) containing 6% of lidocaine (ROX-828) for acute treatment of migraine: safety and efficacy data from a phase II clinical trial. Cephalalgia.

[31] Schaffer JT, Hunter BR, Ball KM, Weaver CS (2015). Noninvasive sphenopalatine ganglion block for acute headache in the emergency department: a randomized placebo-controlled trial. Ann Emerg Med.

[32] Cady RK, Saper J, Dexter K, Cady RJ, Manley HR (2015). Long-term efficacy of a double-blind, placebo-controlled, randomized study for repetitive sphenopalatine blockade with bupivacaine vs. saline with the Tx360 device for treatment of chronic migraine. Headache.

[33] Saade E, Paige GB (1996). Patient-administered sphenopalatine ganglion block. Reg Anesth.

[34] Costa A, Pucci E, Antonaci F, Sances G, Granella F, Broich G (2000). The effect of intranasal cocaine and lidocaine on nitroglycerin-induced attacks in cluster headache. Cephalalgia.

[35] Raj PLL, Erdine S (2003). Radiographic imaging for regional anesthesia and pain management.

[36] Levin M (2010). Nerve blocks in the treatment of headache. Neurotherapeutics.

[37] Mohammadkarimi N, Jafari M, Mellat A, Kazemi E, Shirali A (2014). Evaluation of efficacy of intra-nasal lidocaine for headache relief in patients refer to emergency department. J Res Med Sci.

[38] Kudrow L, Kudrow DB (1995). Intranasal lidocaine. Headache.

[39] Maizels M (1999). Intranasal lidocaine to prevent headache following migraine aura. Headache.

[40] Saberski L, Ahmad M, Wiske P (1999). Sphenopalatine ganglion block for treatment of sinus arrest in postherpetic neuralgia. Headache.

[41] Chae HSJ, Nguyen M, Lee A (2006). The use of intranasal sphenopalatine ganglion blockade for the treatment of post-traumatic headache: a case series. Arch Phys Med Rehabil.

[42] Cohen S, Sakr A, Katyal S, Chopra D (2009). Sphenopalatine ganglion block for postdural puncture headache. Anaesthesia.

[43] Bakbak B, Gedik S, Koktekir BE, Okka M (2012). Cluster headache with ptosis responsive to intranasal lidocaine application: a case report. J Med Case Rep.

[44] Candido KD, Massey ST, Sauer R, Darabad RR, Knezevic NN (2013). A novel revision to the classical transnasal topical sphenopalatine ganglion block for the treatment of headache and facial pain. Pain Physician..

[45] Cohen S, Ramos D, Grubb W, Mellender S, Mohiuddin A, Chiricolo A (2014). Sphenopalatine ganglion block: a safer alternative to epidural blood patch for postdural puncture headache. Reg Anesth Pain Med.

[46] Dance LAD, Schaefer C, Kaye R, Yonker M, Towbin, R (2017) Safety and efficacy of sphenopalatine ganglion blockade in children – initial experience. Journal of Vascular and Interventional Radiology 28:2(Supplement S8).

